# Aboutness in imagination

**DOI:** 10.1007/s11098-017-0937-y

**Published:** 2017-06-07

**Authors:** Francesco Berto

**Affiliations:** Department of Philosophy, Institute for Logic, Language and Computation (ILLC), University of Amsterdam, Oude Turfmarkt 141-147, 1012 GC Amsterdam, Netherlands

**Keywords:** Aboutness, Content-preserving entailment, Logic of imagination, Intentionality

## Abstract

I present a formal theory of the logic and aboutness of imagination. Aboutness is understood as the relation between meaningful items and what they concern, as per Yablo and Fine’s works on the notion. Imagination is understood as per Chalmers’ *positive conceivability*: the intentional state of a subject who conceives that *p* by imagining a situation—a configuration of objects and properties—verifying *p*. So far aboutness theory has been developed mainly for linguistic representation, but it is natural to extend it to intentional states. The proposed framework combines a modal semantics with a mereology of contents: imagination operators are understood as variably strict quantifiers over worlds with a content-preservation constraint.

## Features of aboutness

*Aboutness* is “the relation that meaningful items bear to whatever it is that they are *on* or *of* or that they *address* or *concern*” (Yablo 2014, p. 1).^1^ Research on aboutness has been flourishing, mainly thanks to the works of Yablo and Kit Fine [see e.g. Fine (2014, 2015)]. However, before the introduction of the “grand-sounding name for something basically familiar” (Yablo 2014, p. 1), logicians and semanticists had already been looking for content-preserving entailment relations holding between two meaningful items *A* and *B* only when *B* introduces no content alien to what *A* is about. Among such relations are those labeled as “tautological entailment” (Van Fraassen 1969), “analytic containment” (Angell 1977; Correia 2004), “analytic implication” (Parry 1933; Fine 1986, 2015; Ferguson 2015).

Work on the topic has clarified a number of points. Firstly, if what a sentence is about can be (properly) included in what another one is about, then contents should be capable of standing in mereological relations [see Yablo (2014, Section 2.3), Fine (2015, Sections 3, 4, 5)]. They should be capable of being fused into wholes which inherit the proper features from the parts [see Yablo (2014, Section 3.2)].

Secondly, we have paradigmatic cases of content inclusion and noninclusion already at the propositional level. These concern conjunction and disjunction, and have been taken as data a theory of content-preserving entailment must comply with:

A paradigm of inclusion, I take it, is the relation that simple conjunctions bear to their conjuncts – the relation *Snow is white and expensive* bears, for example, to *Snow is white*. A paradigm of noninclusion is the relation disjuncts bear to disjunctions; *Snow is white* does not have *Snow is white or expensive* as a part. (Yablo 2014, p. 11)

A guiding principle behind the understanding of partial content is that the content of *A* and *B* should each be part of the content of $A \wedge B$ but that the content of $A \vee B$ should not in general be part of the content of either *A* or *B*. (Fine 2015, p. 1)

Thirdly, a negative point of agreement is that standard possible worlds semantics alone is not suitable for modeling aboutness-preserving entailment. If we understand the content of a sentence just as a set of possible worlds, we will have no hint on how and why the sentence should be true at those worlds. There is broad agreement that the right semantics should be hyperintensional [see Yablo (2014, p. 62)]: it should be able to draw distinctions between necessarily equivalent contents, whereas standard possible worlds semantics only draws intensional distinctions [see Jago (2014)]. Even though “$7 + 5 = 12$” and “$x^{n} + y^{n} = z^{n}$ has no solutions in positive integers for $n > 2$” are true in the same possible worlds, they seem to be about different things. Relatedly, the notion of logical consequence captured in a classical or (normal) modal setting is not aboutness-preserving.^2^

## Aboutness and imagination

While the work of Yablo and Fine addresses aboutness mainly as a feature of linguistic representations, another kind of representation bears aboutness, too: mental representation.^3^ Brentano may have been wrong in claiming that all mental states bear intentionality, but most scholars agree that some do, and “every intentional state or episode has an object—something it is about or directed on” (Crane 2013, p. 4).

The goal of this paper is to investigate the logic and aboutness of a kind of intentional states, often gathered under the labels of “imagination” and “conceivability”. Specifically, I rely on a notion one can find in the works of Yablo himself, and Chalmers (Yablo 1993; Chalmers 2002), and dubbed by the latter “positive conceivability”. Positively conceiving that S is understood as a mental operation different from merely supposing or assuming that S, as when we make an assumption in a mathematical proof. Instead, we represent a situation in our mind—a configuration of objects and properties of which S is a truthful description:

Positive notions of conceivability require that one can form some sort of positive conception of a situation in which S is the case. One can place the varieties of positive conceivability under the broad rubric of *imagination*: to positively conceive of a situation is to imagine (in some sense) a specific configuration of objects and properties. [...] Overall, we can say that S is positively conceivable when one can imagine that S: that is, when one can imagine a situation that verifies S. (Chalmers 2002 p. 150)

This seems to be a notion typically at issue in debates on whether conceivability entails possibility [see e.g. Hill (1997), Gendler (2000), Bealer (2002), Currie (2002), Stoljar (2007), Kung (2010), Roca-Royes (2011), the essays in Gendler and Hawthorne (2002)].^4^ I take it that positive conceivability or imagination, so understood, has a number of features any acceptable modeling of it must comply with. And such features bring to the fore the connections between imagination and aboutness theory.

Firstly, the aboutness of imagination ought to be hyperintensional, too, drawing distinctions between necessarily or logically equivalent contents. Arguably, Lois Lane can imagine that Superman is in love with her without imagining that Clark Kent is in love with her, and we can conceive that $7 + 5 = 12$ without conceiving that Fermat’s Last Theorem is true.^5^ Thus, it is difficult to model it via standard possible worlds semantics. But things may improve when we add a proper account of aboutness and content-inclusion. This is the route pursued in some formal detail below.

It is also the route followed in (Yablo 2014, Ch. 7) for a different intentional state: knowledge. It seems that knowledge ought to be hyperintensional because of putative counterexamples to its Closure principle: if *A* is known, and *A* entails *B*, then *B* should also be known, or at least no less knowable. Closure is an automatic spin-off of the modeling of knowledge as a restricted quantifier over possible worlds in standard epistemic logic from Hintikka (1962) onwards. But Yablo suggests that, although knowledge may fail to be closed under an ampliative notion of entailment such as the one captured in standard possible worlds semantics, we can save the idea that knowledge is closed under an aboutness-preserving notion of entailment: if *x* knows that *A* and *B*’s content is part of that of *A*, then *x* is in a position to know *B* as well. Below, I will defend the view that something similar holds for imagination—of the kind we are investigating.^6^

Secondly, and more generally, there is indeed such a thing as a *logic* of imagination. This may sound controversial to one who has a generic understanding of imagination as anarchic mental wandering – a *tonk*-like runabout inference ticket (as the motto has it, “Logic will get you from *A* to *B*; imagination will take you everywhere”). Whether or not there is such a thing as fully anarchic imagination, it is specifically with imagination as positive conceivability that we are dealing with. So understood, that is, as mental representation of a situation verifying some claims, the activity is logically constrained. We simulate alternatives to reality in our mind, in order to explore what would and would not happen if they were realized [this can often help us to cope with reality itself, by improving future performance, allowing us to make contingency plans, etc.—see e.g. the works in Markman et al. (2009)]. That some things would happen in the envisaged scenario, and some would not, means that such exercises, as persuasively argued by Byrne (2005), have a logic: some things follow in the conceived situation, some do not. As we will see below, a logic of aboutness—or content-containment can capture intuitively valid logical patterns for positive conceivability.

Thirdly, while in imagination we do in a sense less then drawing all the classical logical consequences of what we imagine, in another sense we do more. As stressed e.g. in Wansing (2015), conscious acts of imagination have a deliberate, explicit starting point: we set out to target a chosen content. The explicit input may be made up by the conceiver (“Now let us imagine what would happen if...”), or it may be given as an external instruction (think of going through a novel and take the sentences you read as your input). Also according to the mental simulation model of Nichols and Stich (2003), imagination has a deliberate starting point: “an initial premiss or set of premisses, which are the basic assumptions about what is to be pretended” (ibid, p. 24). However, “children and adults elaborate the pretend scenarios in ways that are not inferential at all”, filling in the explicit instruction with “an increasingly detailed description of what the world would be like if the initiating representation were true” (ibid, pp. 26–28). We integrate the explicit input with background information we import into the scenario, on the basis of what we know or believe. You read a Jeffery Deaver book featuring Lincoln Rhyme, a detective working in New York on some murder case. The sentences of the book give you the explicit input. On this basis, you start imagining the situation. You do it by unpacking information which is in some sense included, for all you know or believe, in the scenario: New York is in the US, and normally detectives are human beings, although, suppose, the Deaver story does not state these things explicitly. Absent information to the contrary, you take such facts as holding in the imagined situation: you do imagine Lincoln as a human being working in the US, although this is not entailed via sheer logic by the explicit input. I propose to model this feature of imagination via modal operators interpreted as *variably* strict quantifiers over worlds. The variability of strictness accounts for the contextual selection of the information we import in an act of imagination when we integrate its explicit input. As we will see, the input will play a role similar to a variably strict conditional antecedent, in the style of the possible worlds semantics for counterfactuals due to Stalnaker (1968), Lewis (1973).^7^

Fourthly, although in imagination we go beyond the explicit input, what we can have a logic *of* is the part of this going beyond that sticks with what the explicit input is *about*. We do not indiscriminately import arbitrary, unrelated contents into the conceived scenarios: what background is imported is constrained by what is relevant with respect to the explicit input. You know that Manila is the capital of the Philippines, but this is immaterial to your imagining Lincoln Rhyme’s New York adventures as per Deaver’s book, in so far as such adventures do not involve Manila or the Philippines at all. So you will not, in general, import such irrelevant content in your scenario. Of course, you *can* imagine things about Manila as well, by some free-floating association of ideas triggered by the Lincoln Rhyme input. But that is precisely the anarchic aspect of imagination as generally understood, which seems not to be subject to logical regimentation. What can be regimented—or so I will try to show below—is a selection of information based on relevance with respect to the explicit input. I propose that relevance be here understood as aboutness-preservation: the enriched content must be, in the appropriate sense, included in what the explicit input is about.

If this list of requirements is found plausible, we can now make things precise via a formal semantics that fulfills them. Before we start, I should at this point mention the companion paper to the one you are reading, namely Berto (2017), where I present a formal semantics for imagination whose outcomes in terms of valid and invalid entailments are similar to the ones explored below. The framework used there, however, is very different: I employ so-called non-normal or impossible worlds, understood as worlds where logic is different [see Berto (2013), Nolan (2013) for introductions; see also Priest (2001, Chs. 4, 9), Jago (2014)]; and I have no mereology of contents in the semantics. The impossible worlds framework is much more flexible than the one explored below when it’s about making hyperintensional distinctions. However, many don’t like impossible worlds. The semantics below, instead, uses only possible worlds and has a classical (normal modal) notion of consequence. This may be more palatable for conservative logicians and philosophers. I will anyway refer back to Berto (2017) in the coming Sections, for a few comparisons.

## A semantics of imagination

We have a propositional language $\mathcal {L}$ with an indefinitely large set $\mathcal {L}_{AT}$ of atomic formulas, *p*, *q*, *r* ($p_{1}, p_{2}, ...$). We have negation $\lnot$, conjunction $\wedge$, disjunction $\vee$, a conditional $\rightarrow$, square and round brackets, [, ], (, ). We use *A*, *B*, *C*, ..., as metavariables for formulas of $\mathcal {L}$. The well-formed formulas are items in $\mathcal {L}_{AT}$ and, if *A* and *B* are formulas:

$$\begin{aligned} \lnot A \ | \ (A \wedge B) \ | \ (A \vee B) \ | \ (A \rightarrow B) \ | \ [A]B \end{aligned}$$

(Outermost brackets are normally omitted.) We can then identify $\mathcal {L}$ with the set of its well-formed formulas. Expressions of the form “[*A*]” are to be thought of as sententially indexed modal operators [the idea goes back to Chellas (1975)]. We will consider specific *acts of imagination* performed by conceiving agents on specific occasions, and characterized by an explicit input—what the agent sets out to imagine—directly given by a formula of $\mathcal {L}$. If *K* is the set of formulas standing for possible explicit inputs, then for $A \in K$, [*A*] is the corresponding modal. We read “[*A*]*B*” as “It is imagined in the act whose explicit input is *A*, that *B*” or, more tersely, “It is imagined in act *A* that *B*”.^8^

We may or may not want to have $K = \mathcal {L}$ (hence *K* is flagged separately). One may, for instance, take into account finitary constraints on the agent, to the effect that it just cannot explicitly represent contents expressed by formulas above a certain level of logical complexity. How to circumscribe *K* accordingly may be a substantive task, depending on the desired constraints. I just mention that for $K \subset \mathcal {L}$, one would put a corresponding restriction directly in the syntax of the language, allowing “[*A*]*B*” to be well-formed only for $A \in K$.

As for the semantics: if subject matter is “an independent factor in meaning, constrained but not determined by truth conditions” (Yablo 2014, p. 2), then one way to model it is to combine a truth-conditional, possible worlds setting with a structure of contents, as it happens in works on analytic implication such as Urquhart (1973), Fine (1986). A *frame* for $\mathcal {L}$ is a tuple $\mathfrak {F}$ = $\langle W, \{R_{A} \ | \ A \in K\}, \mathcal {C}, \oplus , c \rangle$, understood as follows. *W* is a set of possible worlds. $\{R_{A} \ | \ A \in K\}$ is a set of accessibilities between worlds, where each $A \in K$ has its own $R_{A} \subseteq W \times W$. $\mathcal {C}$ is a finite set of *contents* (finiteness complies with the idea that a real conceiving agent will only have at most a finite amount of concepts at its disposal). Contents are the situations intentional acts of imagination are about. In the metalanguage we use variables $w, w_{1}, w_{2}, ...$, ranging over possible worlds, $x, y, z \ (x_{1}, x_{2}, ...)$, ranging over contents, as well as the symbols $\Rightarrow , \Leftrightarrow , \& , or, \sim , \forall , \exists$, with the usual reading. $\oplus$ is *content fusion*, a binary operation on $\mathcal {C}$ making of contents part of larger contents and satisfying, for all $xyz \in \mathcal {C}$:

- (Idempotence) $$x \oplus x = x$$
- (Commutativity) $$x \oplus y = y \oplus x$$
- (Associativity) $$(x \oplus y) \oplus z = x \oplus (y \oplus z)$$

We assume unrestricted fusion, that is, $\oplus$ is always defined on $\mathcal {C}$: $\forall xy \in \mathcal {C} \ \exists z \in \mathcal {C} (z = x \oplus y)$. We then define *content parthood*, $\le$, the usual way: $\forall xy \in \mathcal {C} (x \le y \Leftrightarrow x \oplus y = y)$. This makes of parthood a partial ordering—for all $xyz \in \mathcal {C}$:

- (Reflexivity) $$x \le x$$
- (Antisymmetry) $$x \le y \ \& \ y \le x \Rightarrow x = y$$
- (Transitivity) $$x \le y \ \& \ y \le z \Rightarrow x \le z$$

Thus, $\langle \mathcal {C}, \oplus \rangle$ is a join semilattice and, because $\mathcal {C}$ is finite, it is also complete: any set of contents $S \subseteq \mathcal {C}$ has a fusion $\oplus S$. We can think of all contents in $\mathcal {C}$ as built via fusions out of atoms, contents with no proper parts: $Atom(x) \Leftrightarrow \ \sim \exists y (y < x)$, with < the strict order defined from $\le$.

Our *c* in $\mathfrak {F}$ above is a function $c: \mathcal {L}_{AT} \rightarrow \mathcal {C}$, such that if $p \in \mathcal {L}_{AT}$, then $c(p) \in \{x \in \mathcal {C} | Atom(x)\}$: atomic contents are assigned to atomic formulas (this is an idealization, for grammatically simple sentences of ordinary language can be about intuitively complex contents; but it will streamline the discussion below). Next, *c* is extended to the whole of $\mathcal {L}$ as follows. if ${\mathfrak{At}}A = \{p_{1}, ..., p_{n}\}$, the set of atoms in *A*, then:

$$\begin{aligned} c(A) = \oplus {\mathfrak{At}}A = c(p_{1}) \oplus ... \oplus c(p_{n}) \end{aligned}$$

Intuitively, a formula is about whatever its atoms taken together are about. This mereology of contents tracks syntactic structure only so far. It entails, by induction on the construction of formulas, not only that $c(A) = c(\lnot \lnot A)$ (recall Frege on the *Sinn*-preservation of Double Negation), but also that $c(A) = c(\lnot A)$: a formula is about what its negation is about. And not only $c(A \wedge B) = c(B \wedge A)$, but also, e.g., $c(A \wedge B) = c(A) \oplus c(B) = c(A \vee B)$. These are often taken as requirements for aboutness—or content-inclusion in the literature [e.g., in Yablo (2014, p. 42), Fine (2015, p. 11)]. As we will see, this will not entail that imagining that $A \wedge B$ is the same as imagining that $A \vee B$. The two acts will be about the same stuff, but the stuff will be imagined in two quite different *ways*.

A frame becomes a *model* $\mathfrak {M}$ = $\langle W, \{R_{A} \ | \ A \in K\}, \mathcal {C}, \oplus , c, \,\Vdash\, \rangle$ when endowed with an interpretation $\,\Vdash \subseteq W \times \mathcal {L}_{AT}$. This relates worlds to atoms: we read “$w \,\Vdash\, p$” as meaning that *p* is true at *w*, “$w \,\nVdash\, p$” as $\sim w\,\Vdash\, p$. Next, $\Vdash$ is extended to all formulas of $\mathcal {L}$ as follows:

- (S$$\lnot$$) $$w \,\Vdash\, \lnot A \Leftrightarrow w \,\nVdash\, A$$
- (S$$\wedge$$) $$w \,\Vdash\, A \wedge B \Leftrightarrow w \,\Vdash\, A \ \& \ w \,\Vdash\, B$$
- (S$$\vee$$) $$w \,\Vdash\, A \vee B \Leftrightarrow w \,\Vdash\, A \ or \ w \,\Vdash\, B$$
- (S$$\rightarrow$$) $$w \,\Vdash\, A \rightarrow B \Leftrightarrow \forall w_{1}(w_{1} \,\Vdash\, A \Rightarrow w_{1} \,\Vdash\, B)$$
- (S[*A*]) $$w \,\Vdash\, [A]B \Leftrightarrow \forall w_{1}(wR_{A}w_{1} \Rightarrow w_{1} \,\Vdash\, B) \ \& \ c(B) \le c(A)$$

Read “$wR_{A}w_{1}$” as meaning that $w_{1}$ is accessed by an act of imagination with explicit input *A*, obtaining at *w*. It is vital that accessibilities be input-indexed: acts with different explicit inputs will have the conceiving agent look at different sets of worlds. (S[*A*]) can be equivalently expressed using set-selection functions [inspired by Lewis (1973)]. Each $A \in K$ has a function $f_{A}: W \rightarrow \mathcal {P}(W)$, taking as input the world where the act obtains and giving as output the set of worlds accessible via that act, $f_{A}(w) = \{w_{1} \in W | wR_{A}w_{1} \}$. If $|A| = \{w \in W | w \,\Vdash\, A\}$, we can compactly rephrase the clause for [*A*] as:

- (S[*A*]) $$w \,\Vdash\, [A]B \Leftrightarrow f_{A}(w) \subseteq |B| \ \& \ c(B) \le c(A)$$

The two formulations are equivalent since $wR_{A}w_{1} \Leftrightarrow w_{1} \in f_{A}(w)$. However, it will sometimes be easier to make a point using either formulation rather than the other. Set-selection functions can also tersely express a natural Basic Constraint on the semantics – that for all $A \in K$ and $w \in W$:

$$\begin{aligned} \hbox {(BC) }f_{A}(w) \subseteq |A| \end{aligned}$$

This is equivalent to $\forall w_{1}(wR_{A}w_{1} \Rightarrow w_{1} \,\Vdash\, A)$, thus BC says that all the *A*-accessible worlds will be *A*-worlds: worlds making the explicit input *A* true. Besides being intrinsically plausible, this will come in handy to prove a number of results below. From now on, we will only consider models satisfying BC.

For [*A*]*B* to come out true we ask, thus, two things at once. Firstly, we have a truth-conditional requirement: that *B* be true throughout a selected set of worlds making (by BC) the explicit input *A* true. Secondly, we have an aboutness requirement: that *B* allows no content alien to *A* to sneak in.

Finally, we define logical consequence the standard way, as truth preservation at all worlds of all (admissible) models. With $\Sigma$ a set of formulas:

$\Sigma \,\vDash\, B \Leftrightarrow$ in all models $\mathfrak {M}$ = $\langle W, \{R_{A} \ | \ A \in K\}, \mathcal {C}, \oplus , c, \,\Vdash\, \rangle$ and for all $w \in W$: $w \,\Vdash\, A$ for all $A \in \Sigma \Rightarrow w \,\Vdash\, B$

For single-premise entailment, we will write $A \,\vDash\, B$ for $\{A\} \,\vDash\, B$. As a special case, logical validity, $\,\vDash\, A$, truth at all worlds of all admissible models, is $\emptyset \,\vDash\, A$, entailment by the empty set of premisses.

The logic induced by the semantics for the extensional operators is just classical propositional, with $\rightarrow$ a strict S5-like conditional. The novelty comes with [*A*]*B*, whose logical and aboutness features we are now going to unpack.

## Imagining parts and wholes

Our first logical validity is:

$$\begin{aligned} \,\vDash\, [A]A \end{aligned}$$

The content of the explicit input is always imagined. This is immediately guaranteed by our Basic Condition.^9^

The next validities show that imagination is “fully conjunctive”. They mirror in the language the proper mereological structure in the aboutness of imagination^10^:

$$\begin{aligned} \hbox {(Simplification) }[A](B \wedge C) \,\vDash\, [A]B \ \ \ [A](B \wedge C) \,\vDash\, [A]C \end{aligned}$$

Thus, imagination complies with Yablo’s “paradigm of inclusion”: the entailment from a conjunction to its conjuncts. The companion of Simplification is^11^:

$$\begin{aligned} \hbox {(Adjunction) }\{[A]B, [A]C\} \,\vDash\, [A](B \wedge C) \end{aligned}$$

Adjunction may look more controversial than Simplification. This point is also discussed in Berto (2017), which adapts an old example by Quine (1960, p. 222) concerning same-antecedent counterfactuals allowing conjunction of their consequents. The explicit input indexing [*A*] involves Caesar being in command of the US troops in the war of Korea. We can imagine him using bombs, *B*, importing in the representation the weapons available in the Korean war, or we can imagine him using catapults, *C*, importing the military apparatus available to Caesar. However, one would not thereby infer $[A](B \wedge C)$, Caesar’s employing both bombs and catapults. One can imagine *that*, too, if one likes, but it should not come out as an automatic entailment from the logic of imagining.

However, Adjunction should be maintained. Acts of imagination are contextually determined: the same explicit input (say, the same short story) can be the starting point of different acts in different circumstances (the time and place, background information, etc.). In the example adapted from Quine there is a clear contextual shift. But once the context is fixed, Adjunction will work. (The formalism may easily represent this directly, if wanted, by adding to our frames a set of contexts and variables ranging over them, indexing representational acts: $[A]_{x}$, $[A]_{y}$ will then stand for two distinct acts with the same explicit input *A*.) Recall that we are not targeting mere supposition, but positive conceivability: one’s imagining is about a situation, a configuration of objects and properties. In Berto (2017), it is argued that such contents have some mereological structure. When one imagines the whole of a situation, one imagines its parts: we cannot imagine that John is an Englishman living in New York without imagining that John is an Englishman and that John lives in New York, for the last two contents are parts of the scenario we are imagining. Vice versa, when we imagine that John is an Englishman and that John lives in New York in the very same imaginative act (i.e., modulo the fixing of context), we imagine the whole scenario.

It should be stressed that the conjunctive features of imagination are embedded in our semantics with possible worlds and content-containment, but they are not inevitable in the impossible worlds framework of Berto (2017): there, imagination can only be made fully conjunctive by adding specific conditions on impossible worlds frames, which involve the $R_{A}$-accessibilities. If one really does not like imagination to be adjunctive, such conditions can be dropped. This is one way in which the impossible worlds framework for imagination is more flexible than the one explored here.

## Disjunctive and vague imaginings

Yablo’s “paradigm of noninclusion”, that is, of (classically valid) entailment which is not aboutness-preserving, is the entailment from a formula to a disjunction between it and something else. This needs to fail for the aboutness of imagination, too. When one imagines in an act whose explicit input is *A*, that *B*, one does not thereby imagine a disjunction between the latter and an unrelated *C*. Thus we need, and we get^12^:

$$\begin{aligned}{}[A]B \,\nvDash\, [A](B \vee C) \end{aligned}$$

Notice that the inference fails for the right reason: although $A \,\vDash\, A \vee B$, disjunction can bring in alien content. Another disjunction-involving issue has to do with the fact that imagination generally under-determines its contents. We imagine things vaguely, without this entailing that we imagine vague things. You imagine the crowded streets of New York and you think about a complex scenario involving cabs running around, people in restaurants, skyscrapers, etc. You do not imagine all the details, but you want the details to be there, so to speak. Although you do not imagine the city building by building, New York is not a vague object in your scenario, one with an objectively indeterminate number of buildings. Either the number of buildings of New York is odd, or it is even. But you do not imagine it either way. So we need, and we get^13^:

$$\begin{aligned}{}[A](B \vee C) \,\nvDash\, [A]B \vee [A]C \end{aligned}$$

Here, too, the inference fails for the right reason. Aboutness-inclusion works: when one imagines that either *B* or *C*, one’s imaginative act is about both, just as when one imagines that *B* and that *C* ($c(B \wedge C) = c(B) \oplus c(C) = c(B \vee C)$). However, conjunctive and disjunctive imaginings are different ways of imagining, in that the latter hosts indeterminacy. When you vaguely imagine John as being left—or right-handed, different worlds you have access to via imagination will fill in the unspecified details in different ways: some will have him left-handed, some right-handed. So you neither determinately imagine him left-handed, nor determinately imagine him right-handed.

## Nonmonotonic, relevant imagination

Our operators are nonmonotonic in the sense of this invalidity^14^:

$$\begin{aligned}{}[A]B \,\nvDash\, [A \wedge C]B \end{aligned}$$

Aboutness is preserved here, for in general if $c(B) \le c(A)$, then also $c(B) \le c(A) \oplus c(C) = c(A \wedge C)$. What does the trick is the variability in strictness of our operators: $f_{A}(w)$ need not be the same as $f_{A \wedge C}(w)$. Acts of imagination with different explicit input have us look at different worlds, for they trigger the importation of different background information. As you imagine that John walks across New York, you imagine him in the US. But if you imagine John walking across New York and that the city has been displaced to Canada, you will not imagine him in the US.

Another invalidity displays the hyperintensionality of imagination^15^:

$$\begin{aligned} A \rightarrow B \,\nvDash\, [A]B \end{aligned}$$

Remember that the arrow is a strict S5-like conditional: when it is true, there is just no way for its antecedent to be true while the consequent is false. But even when all the *A*-worlds are *B*-worlds, thus all the *A*-selected *A*-worlds are *B*-worlds, aboutness-inclusion can fail. *B* may be about things *A* is not about although there is no way for *A* to be true while *B* isn’t. In particular, “[necessary] equivalents are liable to differ in what they’re about, which can drive a wedge between them epistemologically” (Yablo 2014, p. 120).

By being hyperintensional, imagination also has an element of relevance [in the sense of Anderson and Belnap (1975), Anderson et al. (1992)’s relevant logic.] We have an irrelevant strict conditional^16^:

$$\begin{aligned} \,\vDash\, A \wedge \lnot A \rightarrow B \end{aligned}$$

However, the following ensures that we do not conceive arbitrary irrelevant contents just because we conceive a logical inconsistency^17^:

$$\begin{aligned} \,\nvDash\, [A \wedge \lnot A]B \end{aligned}$$

Although there is no possible world where a contradiction is true, when we think about a contradiction we still think about something. In general $A \wedge \lnot A$ is not contentless: its content is whatever *A* is about, and this may not include the content of *B* (*Snow is white and not white* is about snow’s being white, not about grass’ being purple). For the same reason, this *is* a valid entailment:

$$\begin{aligned} \,\vDash\, [A \wedge \lnot A]A \end{aligned}$$

When we imagine that snow is white and not white, our imagining is about snow’s whiteness.^18^

The dual scenario is perhaps less intriguing. It is good that we do not imagine irrelevant logical validities when we imagine something^19^:

$$\begin{aligned} \,\nvDash\, [A](B \rightarrow B) \end{aligned}$$

However, we imagine all aboutness-preserving logical validities that comply with the explicit input, for instance:

$$\begin{aligned}&\,\vDash\, [A](A \rightarrow A)\\&\,\vDash\, [A](A \vee \lnot A) \end{aligned}$$

This is perhaps the point where our models depart from intuitive plausibility. It might be that the best way to get rid of these, if one does not like them, is by having impossible worlds and allowing direct access to them to our acts of imagination.

## Equivalents in imagination

Even without impossible worlds, so far our logic of imagination is relatively weak. This has to do with imagination combining a number of tall orders: the demands of aboutness-preservation, under-determinacy, context-dependence and variability in the selection of imaginatively accessible worlds. However, we can add a constraint on models, whose effect is to limit imagination’s hyperintensionality. In Berto (2017), this is called the Principle of Imaginative Equivalents. For all $A, B \in K$ and $w \in W$:

$$\begin{aligned} \hbox {(PIE) }f_{A}(w) \subseteq |B| \ \& \ f_{B}(w) \subseteq |A| \Rightarrow f_{A}(w) = f_{B}(w) \end{aligned}$$

When all the selected *A*-worlds make *B* true and vice versa, *A* and *B* are equivalent in the sense that, when we imagine either, we look at the same set of worlds.^20^ PIE makes this valid^21^:

$$\begin{aligned} \hbox {(Substitutivity) }\{[A]B, [B]A, [A]C\} \,\vDash\, [B]C \end{aligned}$$

Substitutivity says that “equivalents in imagination” *A* and *B* can be replaced *salva veritate* as modal indexes in $[\ \cdot \ ]$. This seems right, in spite of the many hyperintensional distinctions we may draw in our mind. For suppose that *bachelor* and *unmarried man* are for you equivalent in imagination: you are so firmly aware of their meaning the same, that you cannot imagine someone being one thing without imagining him being the other ($[A]B \ \& \ [B]A$ entails $c(A) = c(B)$: equivalents in imagination are always about the same thing for the conceiving subject). Thus, [*A*]*B*, when you imagine that John is unmarried, you imagine that he is a bachelor, and [*B*]*A*, when you imagine that John is a bachelor, you imagine that he is unmarried. Suppose [*A*]*C*: as you imagine that John is unmarried, you imagine that he has no marriage allowance. Then the same happens as you imagine that he is a bachelor, [*B*]*C*.

Although transitivity fails in general for our operators as a consequence of their aforementioned non-monotonicity, PIE vindicates a Special Transitivity principle (again, similarly to what happens with counterfactuals in the Lewis-Stalnaker conditional logics)^22^:

$$\begin{aligned} \hbox {(ST) }\{[A]B, [A \wedge B]C\} \,\vDash\, [A]C \end{aligned}$$

This may be good or bad for PIE, depending of what we make of ST. It has good instances. [*A*]*B*: as you imagine that John has won the lottery, you imagine that he has a lot of money. $[A \wedge B]C$: as you imagine that John has won the lottery and has a lot of money, you imagine that he is to pay substantive amounts of taxes. Thus, [*A*]*C*: as you imagine that John has won the lottery, you imagine that he is to pay substantive amounts of taxes. It may be, however, that there are intuitive counterexamples to ST, forceful enough to lead us to reject PIE. I should stress that the proof of ST via PIE in Berto (2017) makes essential use of the same conditions which ensure the validity of Simplification and Adjunction in that framework. Those conditions, as I said above, can be dropped in the impossible worlds setting, which again proves more flexible than the one employed here: there, one can retain PIE without ST by dropping the mereological-conjunctive conception of imagination.

## Directions of further inquiry

The proposed framework can be developed in various ways. The first obvious direction of further work would consist in coming up with a proof system sound and complete with respect to the above semantics—or, variations thereof. One variation on the semantics could go as follows. As the models are now, contents are world-invariant, thus aboutness-inclusions and exclusions are necessary. However, one could have different contents assigned to formulas of $\mathcal {L}$ at different worlds. Then instead of a single content-assigning function *c*, one may have for each $w \in W$ its $c_{w}$ and its set of contents $\mathcal {C}_{w}$. One would then have to make decisions on the relations between contents of $R_{A}$-accessible worlds. One plausible constraint will be an anti-monotonicity requirement whereby for all $A \in K$, $wR_{A}w_{1} \Rightarrow \mathcal {C}_{w_{1}} \subseteq \mathcal {C}_{w}$, that is, all $w_{1}$-contents are *w*-contents whenever there is some imaginative accessibility from the latter to the former [compare Fine (1986, p. 171)].

Another variation can be obtained by dropping the idealizing requirement that atomic formulas be assigned atomic contents. As Yablo claimed: “In an ideal language, simple sentences would be true for simple reasons, and complex sentences for complex reasons. In English, simple sentences can be true for complex reasons” (Yablo 2014, p. 59). However, the arguments for validities and invalidities involving imagination operators above would largely go through untouched.

A more interesting direction of inquiry is to go first-order. As the semantics is now, we think of contents as situations and fusions thereof. In a first-order semantics, contents could just be objects and concepts. An atomic formula would have as contents both objects—what its singular terms are about—and concepts—what its predicate is about. We could also have mereological fusions of objects, and mereological fusions of concepts into (structured) concepts. Since we are modeling conceivability and imagination, though, the interesting puzzle to deal with now would be Frege’s: how to make of *Superman is a superhero* and *Clark Kent is a superhero* different contents if we can imagine the former but not the latter, although the object they involve is the same.

Finally, I should mention that one may explore two-place modal operators interpreted as variably strict quantifiers over worlds with content—or topic-preservation constraints, in a general setting. By varying the features of $R_{A}$-accessibilites, and/or by relaxing those constraints (e.g., from full content inclusion to mere content overlap), one may have a whole family of intentional, doxastic or epistemic operators with interesting features and intuitive interpretation.

## Footnotes

1 Between 2016 and 2017, ideas included of this paper have been presented at the workshop *Imagination and Mental Imagery in Epistemology*, University of Antwerp; at the Seminar of the Arché Logic Group, University of St Andrews; at the Tokyo Forum for Analytic Philosophy, University of Tokyo; at the Center for Logic Language and Cognition, University of Turin; and at the Logic of Conceivability Seminar at the University of Amsterdam. I am very grateful to all those who provided comments, including two anonymous referees of this Journal. Special thanks go to the Logic of Conceivability gang, in particular to Chris Badura, Ilaria Canavotto, Jorge Ferreira, Peter Hawke, and Tom Schoonen.

2 Yablo and Fine have different attitudes towards possible worlds semantics: the former retains a possible worlds framework in his book, characterizing *subject matters*—what sentences are about—as certain divisions of the space of worlds. The latter dispenses with worlds altogether in his works, resorting to a state space or a space of truthmakers which can be fused into further truthmakers. We follow an intermediate approach below: we have possible worlds, but we understand aboutness also in terms of contents and mereological relations between them.

3 Actually (Yablo 2014, Ch. 7) goes into the aboutness of epistemic states. I will come to this below.

4 Some philosophers such as, famously, Descartes, made a lot of a distinction between having the concept of something and having a mental image of it. However, in this paper I will use “conceiving” and “imagining” broadly as synonyms for the act of representing a situation making a sentence or proposition true. Such terminological interchangeability seems to be widespread in the literature on the subject, as one can see e.g. in the Chalmersian quote above. In such contexts “imagination” is normally understood broadly enough: imagined contents need not be perforce visually imaginable for they can, for instance, include abstract objects and properties. This is a point stressed also by Williamson (2007), who makes of imagination a key notion in his counterfactual epistemology of metaphysical modality. In Berto and Schoonen (2017), it is conjectured that, from the viewpoint of cognitive psychology, the Yablovian-Chalmersian notion of conceivability is somewhat ambiguous between the having of linguistic-amodal mental representation, and the having of pictorial representation taken as mental imagery. Such ambiguity is important for issues in modal epistemology, but will not be dealt with in this paper. On the topic, see also Balcerak Jackson (2016).

5 That imagination or conceivability *is* hyperintensional in this way is not completely uncontroversial, and some advocates of the entailment from conceivability to possibility may want to deny it. I will not defend the view in this paper. Sustained arguments for the claim that we can conceive impossible situations can be found in Priest (2005), Byrne (2007), Fiocco (2007), Jago (2014), Kung (2014), Berto and Schoonen (2017). But even those who, like Chalmers himself, want to defend the idea that a certain kind of conceivability entails a certain kind of possibility (in the terminology of Chalmers (2002): primary positive ideal conceivability entails epistemic possibility), usually grant that, because intentional states have hyperintensional features, one needs to modify the standard possible worlds apparatus to account for them. This may be achieved by making of the contents of the relevant states structured Russellian propositions, or sets of possible and impossible worlds, or, as in Chalmers’ own view, structures of Fregean senses: see Chalmers (2011).

6 For a limited denial of Closure that employs a kind of aboutness-containment semantics for epistemic operators, see Hawke (2016).

7 One important difference will be that I will not impose a similarity metric on worlds. How to have one such metric for imagination operators is an open issue of the approach developed in this paper. Other works on the logic of imagination in a modal setting take imagination operators as quantifiers over worlds, e.g., Niiniluoto (1985), Costa Leite (2010), or adopt a neighbourhood semantics, as in Wansing (2015) [see also Olkhovikov and Wansing (2017)]. None of them combines variable strictness and aboutness-inclusion as I will do below.

8 Intentional operators are usually agent-indexed in standard epistemic logics, e.g., $K_{x}A$ = “It is known by cognitive agent *x* that *A*”. In a single-agent setting such as the one we are considering, with an agent representing a scenario in the privacy of its own mind, the index does not do a lot of work and can be omitted.

9
*Proof*: by BC, for any *w* and $w_{1}$, $wR_{A}w_{1} \Rightarrow w_{1} \,\Vdash\, A$, and of course $c(A) \le c(A)$.

10
*Proof*: we do the first one (for the second, replace *B* with *C* appropriately). Let $w \,\Vdash\, [A](B \wedge C)$. By (S[*A*]), for all $w_{1}$ such that $wR_{A}w_{1}$, $w_{1} \,\Vdash\, B \wedge C$, thus by (S$\wedge$), $w_{1} \,\Vdash\, B$. Also, $c(B \wedge C) = c(B) \oplus c(C) \le c(A)$, thus $c(B) \le c(A)$. Then, by (S[*A*]), $w \,\Vdash\, [A]B$.

11
*Proof*: let $w \,\Vdash\, [A]B$ and $w \,\Vdash\, [A]C$, that is, by (S[*A*]): for all $w_{1}$ such that $wR_{A}w_{1}$, $w_{1} \,\Vdash\, B$ and $w_{1} \,\Vdash\, C$, so by (S$\wedge$) $w_{1} \,\Vdash\, B \wedge C$. Also, $c(B) \le c(A)$ and $c(C) \le c(A)$, thus $c(B) \oplus c(C) = c(B \wedge C) \le c(A)$. Thus, $w \,\Vdash\, [A](B \wedge C)$.

12
*Countermodel*: let $W = \{w, w_{1}\}$, $wR_{p}w_{1}$, $w_{1} \,\Vdash\, q$, $c(p) = c(q) \ne c(r)$. Then $c(q) \le c(p)$, so by (S[*A*]), $w \,\Vdash\, [p]q$. But $c(q \vee r) = c(q) \oplus c(r) \nleq c(p)$, thus $w \,\nVdash\, [p](q \vee r)$.

13
*Countermodel*: let $W = \{w, w_{1}, w_{2}\}$, $wR_{p}w_{1}$, $wR_{p}w_{2}$, $w_{1} \,\Vdash\, q$ but $w_{1} \,\nVdash\, r$, $w_{2} \,\Vdash\, r$ but $w_{2} \,\nVdash\, q$, $c(p) = c(q) = c(r)$. Then by (S$\vee$), $w_{1} \,\Vdash\, q \vee r$ and $w_{2} \,\Vdash\, q \vee r$, so for all $w_{x}$ such that $wR_{p}w_{x}$, $w_{x} \,\Vdash\, q \vee r$. Also, $c(q \vee r) = c(q) \oplus c(r) \le c(p)$, thus by (S[*A*]), $w \,\Vdash\, [p](q \vee r)$. However, $w \,\nVdash\, [p]q$ and $w \,\nVdash\, [p]r$ for both *p* and *r* fail at some $R_{p}$-accessible world. Thus by (S$\vee$), $w \,\nVdash\, [p]q \vee [p]r$.

14
*Countermodel*: let $W = \{w, w_{1}\}$, *w* $R_{p}$-accesses nothing, $wR_{p \wedge r}w_{1}$, $w_{1} \,\nVdash\, q$, $c(p) = c(q) = c(r)$. Then $w \,\Vdash\, [p]q$, but $w \,\nVdash\, [p \wedge r]q$.

15
*Countermodel*: let $W = \{w, w_{1}\}$, $wR_{p}w_{1}$, $w \,\nVdash\, p$, $w_{1} \,\Vdash\, q$, $c(p) \ne c(q)$. By the BC, $w_{1} \,\Vdash\, p$. Now $|p| \subseteq |q|$, thus $w \,\Vdash\, p \rightarrow q$. But although $f_{p}(w) \subseteq |q|$, $c(q) \nleq c(p)$, thus $w \,\nVdash\, [p]q$.

16 Trivially: for all *w*, $w\,\nVdash\, A \wedge \lnot A$.

17
*Countermodel*: let $W = \{w\}$, $c(p) \ne c(q)$. By the BC, $f_{p \wedge \lnot p}(w) \subseteq |p \wedge \lnot p| = \emptyset \subseteq |q|$. However, $c(q) \nleq c(p \wedge \lnot p) = c(p) \oplus c(\lnot p) = c(p)$. Thus, by (S[*A*]), $w \,\nVdash\, [p \wedge \lnot p]q$.

18 “$p \ \& \ \lnot p$ tautologically entails *p* but not *q* [...] *q* is not ‘already there’ in $p \ \& \ \lnot p$, the way that *p* appears to be already there.” (Yablo 2014, p. 59)

19
*Countermodel*: Let $W = \{w\}, c(p) \ne c(q)$. Then although (trivially) $f_{p}(w) \subseteq |q \rightarrow q|$, $c(q \rightarrow q) = c(q) \nleq c(p)$. Thus by (S[*A*]), $w \,\nVdash\, [p](q \rightarrow q)$.

20 The Principle Imaginative Equivalents would be automatically satisfied if we imposed the appropriate similarity or plausibility metric on worlds. A similar principle holds in fact (as an anonymous referee pointed out) in conditional logics based on such metrics, like the aforementioned Stalnaker (1968), Lewis (1973).

21
*Proof*: Suppose $w \,\Vdash\, [A]B$, $w \,\Vdash\, [B]A$, $w \,\Vdash\, [A]C$. By (S[*A*]), these entail, respectively, (1) $f_{A}(w) \subseteq |B|$ and $c(B) \le c(A)$, (2) $f_{B}(w) \subseteq |A|$ and $c(A) \le c(B)$, (3) $f_{A}(w) \subseteq |C|$ and $c(C) \le c(A)$. From (1) and (2) we get $f_{A}(w) = f_{B}(w)$ (by the PIE) and $c(A) = c(B)$ (by antisymmetry of content parthood). From these and (3) we get $f_{B}(w) \subseteq |C|$ and $c(C) \le c(B)$. Thus by (S[*A*]), $w \,\Vdash\, [B]C$.

22
*Proof*: suppose (1) $w \,\Vdash\, [A]B$ and (2) $w \,\Vdash\, [A \wedge B]C$. From (1), $\vDash\, [A]A$ and Adjunction we get $w \,\Vdash\, [A](A \wedge B)$, thus, by (S[*A*]), $f_{A}(w) \subseteq |A \wedge B|$ and $c(A \wedge B) \le c(A)$. Also, $w \,\Vdash\, [A \wedge B]A$ (from $\,\vDash\, [A \wedge B](A \wedge B)$ and Simplification) thus, by (S[*A*]) again,$f_{A \wedge B}(w) \subseteq |A|$ and (of course) $c(A) \le c(A \wedge B)$. Thus, $f_{A}(w) = f_{A \wedge B}(w)$ (by the PIE) and $c(A \wedge B) = c(A)$ (by antisymmetry of content parthood). Next, by (2) and (S[*A*]) again, $f_{A \wedge B}(w) \subseteq |C|$ and $c(C) \le c(A \wedge B)$. Therefore, $f_{A \wedge B}(w) = f_{A}(w) \subseteq |C|$ and $c(C) \le c(A) = c(A \wedge B)$. Thus by (S[*A*]) again, $w \,\Vdash\, [A]C$.
